# The first X-ray diffraction measurements on Mars

**DOI:** 10.1107/S2052252514021150

**Published:** 2014-10-21

**Authors:** David Bish, David Blake, David Vaniman, Philippe Sarrazin, Thomas Bristow, Cherie Achilles, Przemyslaw Dera, Steve Chipera, Joy Crisp, R. T. Downs, Jack Farmer, Marc Gailhanou, Doug Ming, John Michael Morookian, Richard Morris, Shaunna Morrison, Elizabeth Rampe, Allan Treiman, Albert Yen

**Affiliations:** a Geological Sciences, Indiana University, 1001 E. 10th Street, Bloomington, IN 47405, USA; b NASA Ames Research Center, USA; c Planetary Science Institute, USA; d inXitu, USA; e University of Hawaii at Manoa, USA; f Chesapeake Energy, USA; g NASA Jet Propulsion Laboratory, USA; h University of Arizona, USA; i Arizona State University, USA; j CNRS, France; k NASA Johnson Space Center, USA; l Lunar and Planetary Institute, USA

**Keywords:** X-ray diffraction, Mars, extraterrestrial mineralogy, Curiosity rover

## Abstract

The Mars Science Laboratory landed in Gale crater on Mars in August 2012, and the Curiosity rover then began field studies on its drive toward Mount Sharp, a central peak made of ancient sediments. CheMin is one of ten instruments on or inside the rover, all designed to provide detailed information on the rocks, soils and atmosphere in this region. CheMin is a miniaturized X-ray diffraction/X-ray fluorescence (XRD/XRF) instrument that uses transmission geometry with an energy-discriminating CCD detector. CheMin uses onboard standards for XRD and XRF calibration, and beryl:quartz mixtures constitute the primary XRD standards. Four samples have been analysed by CheMin, namely a soil sample, two samples drilled from mudstones and a sample drilled from a sandstone. Rietveld and full-pattern analysis of the XRD data reveal a complex mineralogy, with contributions from parent igneous rocks, amorphous components and several minerals relating to aqueous alteration. In particular, the mudstone samples all contain one or more phyllosilicates consistent with alteration in liquid water. In addition to quantitative mineralogy, Rietveld refinements also provide unit-cell parameters for the major phases, which can be used to infer the chemical compositions of individual minerals and, by difference, the composition of the amorphous component.

## Abstract 2

The X-ray diffraction/X-ray fluorescence instrument CheMin on the Curiosity rover is a shoebox-sized device using transmission geometry and an energy-discriminating CCD detector. The instrument has returned the first X-ray diffraction data for soil and drilled samples from Mars outcrops, revealing a suite of primary basaltic minerals, amorphous components and varied hydrous alteration products including phyllosilicates.

## 1. Introduction   

Humankind has studied the heavens for millennia and, until the 1960s, all observations of extraterrestrial bodies were made remotely, with either optical or spectroscopic measurements. Similarly, humankind has studied crystalline materials since the beginning of time, but it was not until the discovery of X-ray diffraction (XRD) that we learned about the ordered atomic arrangements that characterize such solids. Thus, von Laue’s discovery in 1912 opened the door to understanding how crystalline solids are constructed, and it became apparent that X-ray diffraction could provide fundamental information on the nature of solids.

Throughout the 20th century, X-ray diffraction became the *de facto* standard in determining the nature and identity of crystalline solids, and X-ray powder diffraction is now routinely exploited in the identification of minerals in geological materials. X-ray diffraction instrumentation was proposed and ultimately built for extraterrestrial exploration by W. Parrish as early as 1960 [Das Gupta *et al.*, 1966 ▶; references in Blake (2000 ▶)], but it was not until the development of CCD X-ray detectors that it became practical to produce a miniaturized low-power X-ray diffraction instrument. The use of two-dimensional detectors was important in simplifying instrument development and minimizing moving parts, and most modern concepts for miniaturized XRD employ transmission geometry and CCD detectors. Interest in planetary X-ray diffraction experienced a resurgence with renewed studies of the planet Mars, and new instrumental concepts began to appear in the early 1990s. Vaniman *et al.* (1991 ▶) and Blake *et al.* (1992 ▶) proposed similar concepts that included either a position-sensitive or a CCD X-ray detector, and these two teams combined in 1992, ultimately leading to the current CheMin instrument on Mars Science Laboratory (MSL). A number of improvements in instrument design and considerable testing of the CheMin instrument occurred throughout the 1990s, including the use of ray-tracing calculations to optimize geometry (Gailhanou *et al.*, 2006 ▶) and sonic sample vibration to minimize sample preparation requirements [Sarrazin *et al.*, 2005 ▶; also see discussion and references in Blake *et al.* (2012 ▶)], and the CheMin instrument was chosen in 2004 as one of the ten instruments on MSL.

MSL launched from Cape Canaveral, Florida, on 26 November 2011, and landed in Gale crater on the morning of 6 August (EDT) 2012. Since that time, the Curiosity rover has traveled more than 8 km towards Aeolis Mons (informally named Mount Sharp). To date, four different samples have been analysed by CheMin, namely Rocknest, an aeolian bedform that was sampled using the rover’s scoop, and two powdered samples of a mudstone and one of a sandstone, sampled by the rover’s drill. Data for the most recent sample, the sandstone from Windjana, have not been released as of this writing and are not discussed here. In a happy coincidence, CheMin’s first XRD analysis on Mars coincided with the 100th anniversary of the discovery of XRD by von Laue.

## 2. Instrumental details   

**Figure 1:**
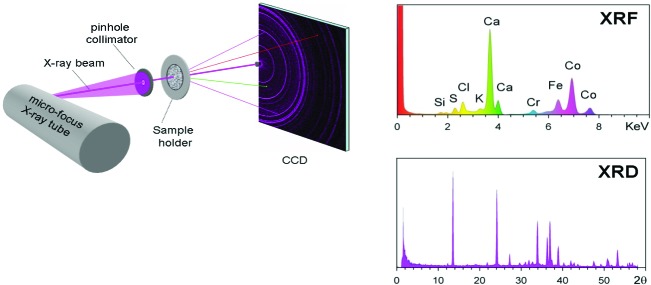
Schematic diagram of the CheMin instrument and resulting XRF and XRD data.

The Mars CheMin instrument is one of ten instruments on the Curiosity rover, which is powered by a radioisotope thermoelectric generator. CheMin (Fig. 1 ▶) uses a microfocus Co X-ray source, producing a collimated ∼70 µm X-ray beam that impinges on a sample held between two polymer windows. The tube produces both characteristic Co radiation and continuum radiation, and a Co anode was chosen to avoid fluorescence encountered with Fe-bearing minerals and Cu radiation. No energy filters are used. CheMin’s detector is a 600 × 1182 pixel E2V CCD224, with 40 µm pixels and a 600 × 582 pixel data-collection area. The CCD is cooled by a cryocooler to below −48°C to reduce dark current, and is operated with a rapid (5–30 s) read-out cycle, allowing single-photon counting (a situation wherein, ideally, zero or one photon strikes each pixel). Operation in single-photon counting mode allows energy discrimination that facilitates resolution of Co *K*α and *K*β photons, for example (and, ideally, eliminates the need for an energy filter). Although large by conventional CCD standards, the 40 µm pixel size reduces the likelihood of charge splitting between pixels, thereby providing more accurate X-ray energy information. Ray-tracing methods were used to optimize the full width at half-maximum (FWHM) and the intensity for the CheMin instrument, which yields one-dimensional XRD patterns with ∼0.3° FWHM (Gailhanou *et al.*, 2006 ▶). Each ‘raw’ frame is measured for 10 s (to ensure single-photon counting), and 180 raw frames are added to create a ‘minor’ frame. The sum of all minor frames for one or more nights of data collection constitutes a ‘major’ frame, and these data are typically used for diffraction analysis (data are collected only at night to obtain the lowest possible CCD temperature, to reduce CCD background noise and to minimize temperature fluctuations over the course of measurement). XRD data presented here were measured over the course of several nights for each sample. X-ray fluorescence analysis is an integral part of the function of the CheMin instrument, as it is a prerequisite for the generation of two-dimensional diffraction patterns from individual wavelengths (*e.g.* Co *K*α 6.925 keV). Thus, it is possible to create two-dimensional images for any wavelength/energy. After producing an *xy* image of all pixels that absorbed a Co *K*α photon (which provides the two-dimensional diffraction patterns shown here), a conventional one-dimensional diffraction pattern is generated using a methodology similar to that employed by *FIT2D* (Hammersley *et al.*, 1996 ▶). More detailed information on the CheMin instrument is included in Blake *et al.* (2012 ▶), along with calibration and test data.

**Figure 2:**
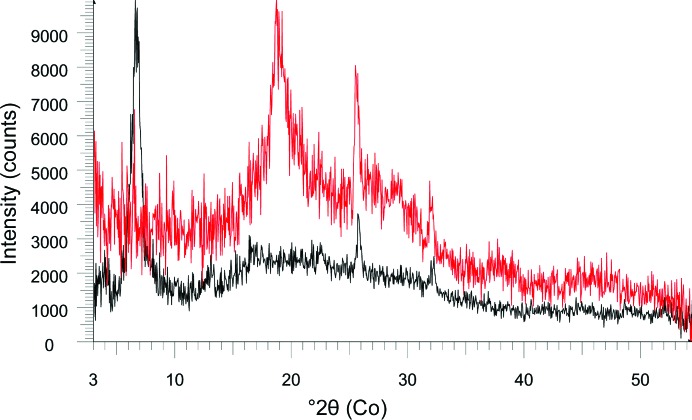
A comparison of XRD patterns from empty Kapton (black) and Mylar (red) cells. Peaks at 25.8 and 32.1° are due to scattering from the Al light shield on the CCD detector.

**Figure 3:**
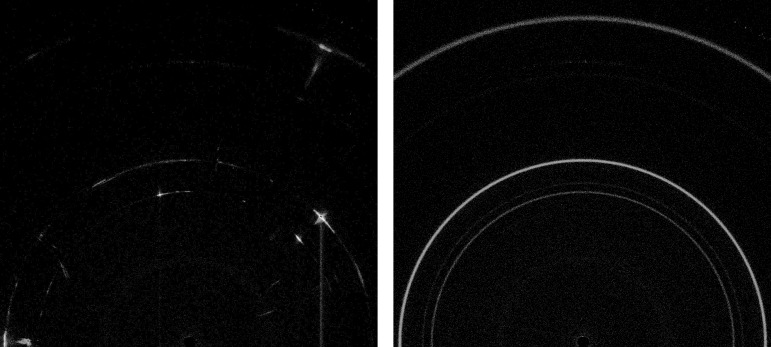
XRD patterns of crushed and sieved (<150 µm) NaCl measured on the CheMin III instrument, (left) without and (right) with sonic vibration.

The sample, sieved to <150 µm by Curiosity’s sample acquisition, sample processing and handling–collection and handling for *in situ* Martian rock analysis (SA/SPaH-CHIMRA) device (Anderson *et al.*, 2012 ▶), is delivered to either 6 µm Mylar or 10 µm Kapton cells, which consist of a sandwich of either polymer, ∼175 µm apart. Fig. 2 ▶ shows a comparison of the scattering from both cells, with a significant peak at ∼6.7° 2θ for Kapton and a broad peak from Mylar at ∼19° 2θ. Although the Kapton cells are more robust than the Mylar cells, they are generally used only when no phyllo­silicates are expected, to avoid the significant low-angle peak from Kapton which would interfere with phyllosilicate 001 reflections. Approximately 10 mm^3^ of sample is required to fill the active volume of the cell, ∼8 mm in diameter. Samples are sonically vibrated at up to 2150 Hz on a tuning fork by piezoelectric actuators to induce flow during analysis, which is very effective in producing acceptable particle statistics and random orientation during analysis, even for unprepared or poorly prepared samples such as those expected on Mars (Sarrazin *et al.*, 2005 ▶). Fig. 3 ▶ shows a comparison of data for an NaCl sample, <150 µm grain size, with and without sonic vibration, illustrating the effectiveness of vibration. The data in Fig. 3 ▶ were measured on Earth using a CheMin III instrument, which is similar to the Mars CheMin instrument.

Gale crater was selected as Curiosity’s landing site for a variety of reasons (Grotzinger *et al.*, 2012 ▶). The crater is nearly 155 km in diameter, ∼4.6 km below the mean average elevation datum, and lies at the equator of Mars, straddling the dichotomy boundary between the northern lowlands and the southern highlands. Because of its latitude, its age (>3.6 Gyr; Wray, 2013 ▶; Le Deit *et al.*, 2013 ▶) and its depth, if Mars had ever had surface water, some of it would have accumulated here. The central mound of Gale is higher than its northern rim and is composed of sediments, including clay minerals, sulfates and oxides. One current hypothesis is that Gale was one of a class of overfilled craters, having been completely filled with sediment that was winnowed out by aeolian processes later in Mars’ history (Grotzinger & Milliken, 2012 ▶). Curiosity landed at the distal end of what was interpreted from orbital data to be an alluvial fan, with deposited sediment transported from Gale’s northern crater wall along what is called Peace Vallis.

**Figure 4:**
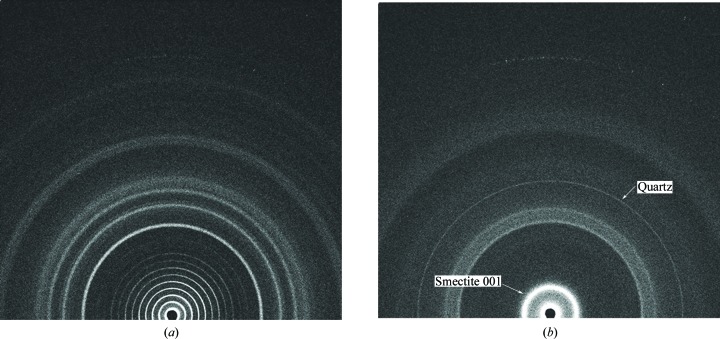
CheMin IV XRD data for (*a*) silver behenate and (*b*) SWa-1 smectite.

The CheMin instrument was designed with the ability to measure data to low angles to evaluate clay mineral diffraction signatures, and Fig. 4 ▶ illustrates the low-angle performance of CheMin III, an early prototype of the CheMin flight instrument. Fig. 4 ▶(*a*) shows a series of higher-order reflections from a *d*(001) of 58.4 Å from silver behenate, with the first peak at 1.75° 2θ (Co). Fig. 4 ▶(*b*) shows the XRD pattern of the SWa-1 ferruginous smectite (Clay Minerals Society Source Clay), with an obvious bright ring due to the 001 smectite reflection at ∼15 Å. Sharper reflections (narrow rings) are due to a small amount of admixed quartz impurity.

### 2.1. Diffraction calibration   

**Figure 5:**
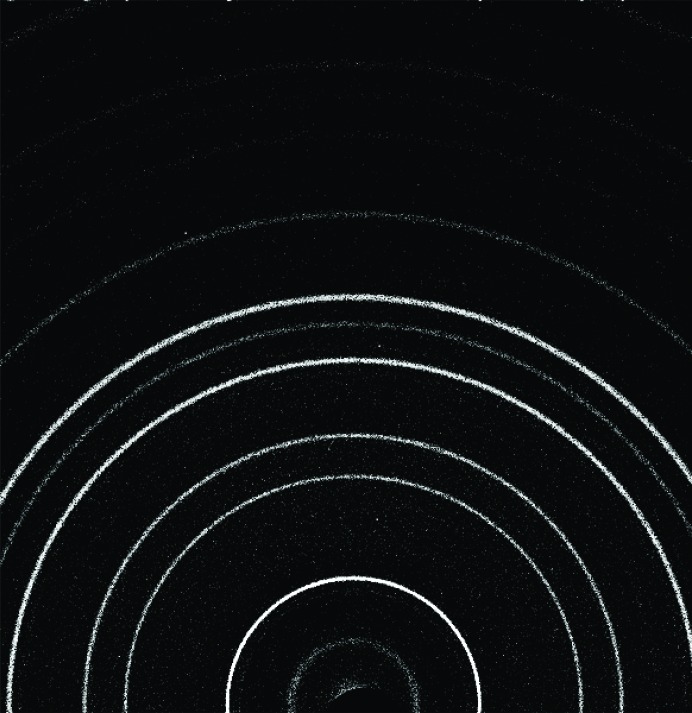
A two-dimensional X-ray diffraction pattern of the beryl:quartz standard measured on Mars.

Several data analysis methods are possible after a major frame is downloaded to Earth. All pixels can be binned to construct an energy histogram, essentially an X-ray fluorescence (XRF) spectrum (*e.g.* the schematic XRF spectrum in Fig. 1 ▶). The XRF data are of only qualitative use, but these data can provide a general chemical picture of the sample and can be useful for detecting small amounts of material remaining in a previously used and dumped sample cell, even when the amounts remaining are too small to produce an XRD pattern (the instrument is largely insensitive to elements below atomic number 19, due to poor CCD quantum efficiency at lower energies and absorption by the Al light shield, the Mars atmosphere and sample self absorption). The analysis procedure for downlinked Mars data first involves visually examining the XRF spectrum to evaluate the quality of the acquired data. In addition to providing a qualitative determination of major elements in the sample, the position and FWHM of the individual maxima (in particular Co *K*α from the X-ray source) provide a figure of merit for the diffraction products. Several two-dimensional diffraction products are downlinked, the most important of which is the ‘energy-filtered diffraction, all’ (EDA), which amounts to a two-dimensional counting number array of the *xy* locations of every Co *K*α X-ray photon absorbed by the CCD. It is equally possible to create a two-dimensional diffraction image using, for example, Co *K*β energies, although the diffracted intensities are lower than for Co *K*α. CheMin carries several pre-loaded sample cells on board for both XRD and XRF standardization, including 45–90 µm mixtures of beryl and quartz (97:3 and 88:12 by weight), pure arcanite (K_2_SO_4_), an amphibole from Gore Mountain, New York, USA, and a synthetic ceramic material of well known chemical composition. These are not typical XRD or XRF standards, but the narrow 2θ range of the CheMin instrument and variable conditions on Mars require unreactive standards with a distribution of significant diffraction peaks from low angle to ∼52° 2θ for Co *K*α (*d* = 1.75 Å). A standard must also be available in a 45–90 µm size fraction and have a low linear absorption coefficient for Co radiation; a beryl:quartz mixture fits these requirements well. Conversely, typical XRD standards such as Si or LaB_6_ have insufficient diffraction peaks in this angular region, and the NIST standards are finely powdered and agglomerate and do not move freely in the CheMin sample holder. In addition, LaB_6_ is insufficiently transparent to Co X-rays (μ > 1700 cm^−1^). Other materials such as alumina were found to be unsuitable in mixtures because significant differences in density gave rise to large amounts of segregation. XRD and XRF data were measured on all of these standards before launch, and the beryl:quartz mixtures were used as the primary XRD standards on Mars. The 88:12 beryl:quartz mixture was first measured on sol 122 (a ‘sol’ is a Martian day) to provide a baseline for the calculation of one-dimensional diffraction patterns from the two-dimensional image shown in Fig. 5 ▶. The commercial program *FilmScan* (MDI Inc., Pleasanton, California, USA) was originally used for these conversions, but we have since adopted more flexible programs, including *GSE_ADA NASA* (Dera *et al.*, 2013 ▶) and *GSASII* (Toby & Von Dreele, 2013 ▶), which accommodate detector tilt more accurately and utilize all of the diffracted peaks to calibrate the pattern. These two-dimensional to one-dimensional routines use data from standard(s) with known unit-cell parameters to refine the sample-to-detector distance and other detector parameters. The sol 122 beryl:quartz data were used, with the unit-cell parameters for beryl and quartz measured on Earth, to determine the important sample-related and instrumental geometry parameters used in the two-dimensional to one-dimensional conversion.

## 3. X-ray diffraction measurements on Mars   

Four distinct powder samples have been analyzed as of this writing, including a scooped sample from an aeolian deposit called Rocknest and two drill samples from a mudstone, called John Klein and Cumberland. Curiosity’s drill uses a 1.6 cm diameter bit, and all powders are sieved to <150 µm by the SA/SPaH-CHIMRA device (Anderson *et al.*, 2012 ▶). In addition, a drill sample referred to as Windjana was obtained from a location known as Kimberley, but results from this sample have not been released and so are not included here.

### 3.1. Rocknest   

**Figure 6:**
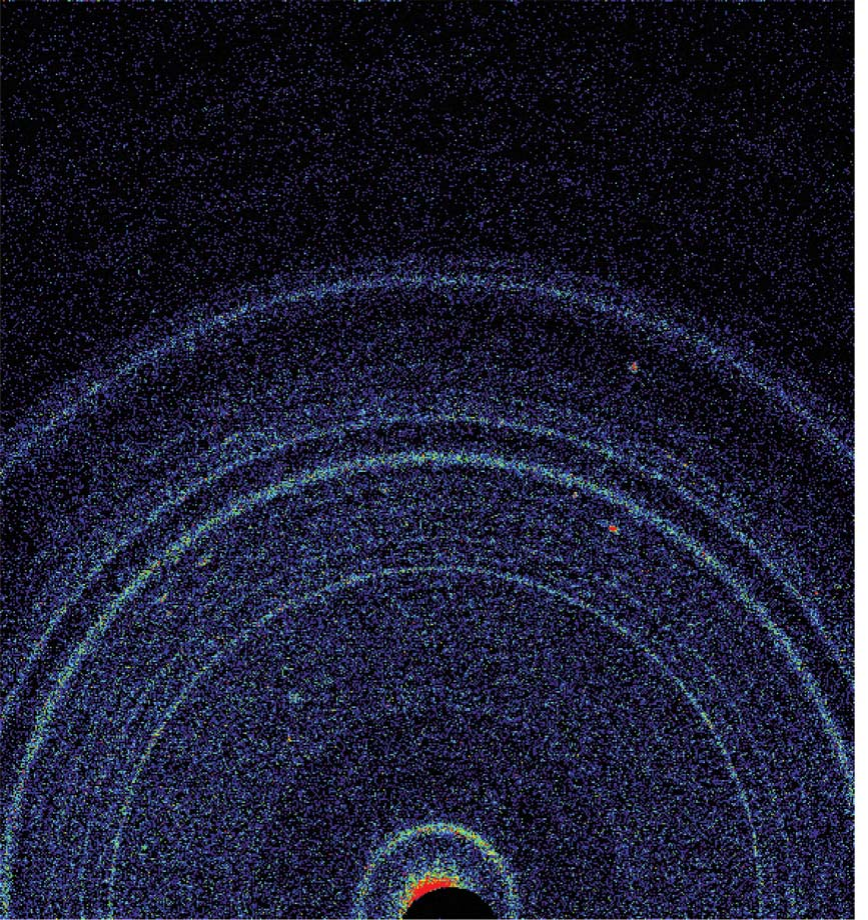
A two-dimensional XRD pattern for the Rocknest aeolian bedform (dune).

**Figure 7:**
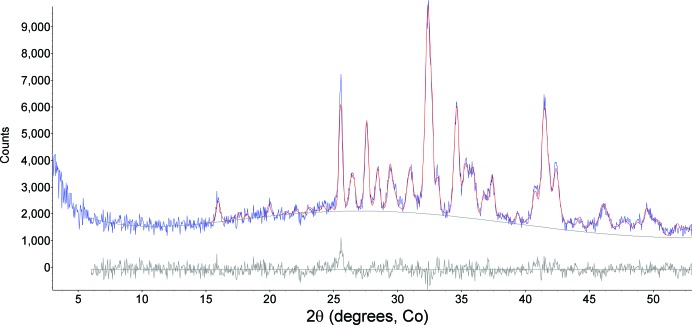
Observed (blue, integrated from the two-dimensional image in Fig. 6 ▶) and calculated (red) plots from Rietveld refinement using data for Rocknest (∼26.4 h integration, phases listed in Table 1 ▶). The calculated background (black line) is inscribed at the base of the observed pattern and the gray pattern at the bottom is the difference plot (observed − calculated). The difference at ∼25.8° is due to scattering from the Al light shield on the CCD.

Curiosity delivered three different scoops of the <150 µm size fraction of the Rocknest material to the CheMin instrument, and Fig. 6 ▶ shows a colorized version of the two-dimensional image for data from the last scoop, representing 26.4 h of integration time (over several nights) in a Mylar cell. The bright bloom at the bottom center of the image is the direct beam, spilling over the beam stop, which is the adjacent black circle. The conventional ‘one-dimensional’ XRD pattern for this sample, along with the fit from the Rietveld refinement, is shown in Fig. 7 ▶; these data were first used to determine the mineralogy of the sample by comparison with data from the powder diffraction file (ICDD, PDF-2, Release 2010). The data were then used in a Rietveld refinement to determine phase abundances and unit-cell parameters for the major phases. Unit-cell parameters were used with published data for the crystalline phases in Table 1 ▶ to determine the chemistry of individual phases (Bish *et al.*, 2013 ▶). All Rietveld refinements noted here used the program *TOPAS* (Bruker AXS), with fundamental parameter profiles determined using the beryl:quartz data from Mars. As noted above, there is a significant contribution at ∼25.6° 2θ from the Al light shield on the CCD, along with a minor contribution from the Mylar cell centered at ∼19°, shown by its contribution to the difference curve in the final Rietveld plot. The broad contributions from the polymer cells are generally minimal in cells containing sample, due to absorption by the sample. These data were fitted well using a model including the phases listed in Table 1 ▶. However, the data in Fig. 7 ▶ show an apparent rise in background throughout the mid-2θ range, due to admixture with an amorphous component(s). The pattern was thus also analyzed using the *FULLPAT* program (Chipera & Bish, 2002 ▶). *FULLPAT* uses a combination of observed data, including those for disordered and amorphous phases, to fit the observed pattern shown here. We used a measured pattern from a basaltic glass to model the amorphous contribution seen in this pattern, giving the results shown in Table 1 ▶.

The quantitative mineralogical results in Table 1 ▶, combined with refined unit-cell parameters for the major phases, facilitated determination of the cumulative composition of the crystalline components. For example, the refined unit-cell parameters for the olivine group mineral, Fe-forsterite, were *a* = 10.327 (7) Å, *b* = 6.034 (7) Å and *c* = 4.771 (5) Å (Bish *et al.*, 2013 ▶). These refined values were used with published information on the olivine minerals to estimate the chemical composition, ∼62% forsterite (*i.e.* Mg_1.24_Fe_0.76_SiO_4_). A similar approach was taken with all other major crystalline phases (Morrison *et al.*, 2013 ▶). This information was then combined with data on the bulk composition determined with the α-particle X-ray spectrometer (APXS) on MSL to obtain information on the composition of the amorphous component(s) [see Blake *et al.* (2013 ▶) for a detailed discussion]. This approach was also used to obtain an independent chemically derived estimate of the amount of the amorphous component(s), which gave a value of 45 wt% amorphous material. Although this value matches the XRD-determined amount within error, the difference may be attributed to cumulative errors in the unit-cell parameter-determined values for individual phase compositions. The CheMin-determined mineralogy of the Rocknest material is similar to the normative mineralogy of other basaltic rocks on Mars and of basaltic Martian meteorites (Treiman *et al.*, 2013 ▶). Perhaps the most interesting aspect of these results is the presence of major amounts of amorphous material, consistent with the lack of significant long-term interactions with liquid water.

### 3.2. Sheepbed mudstone   

**Figure 8:**
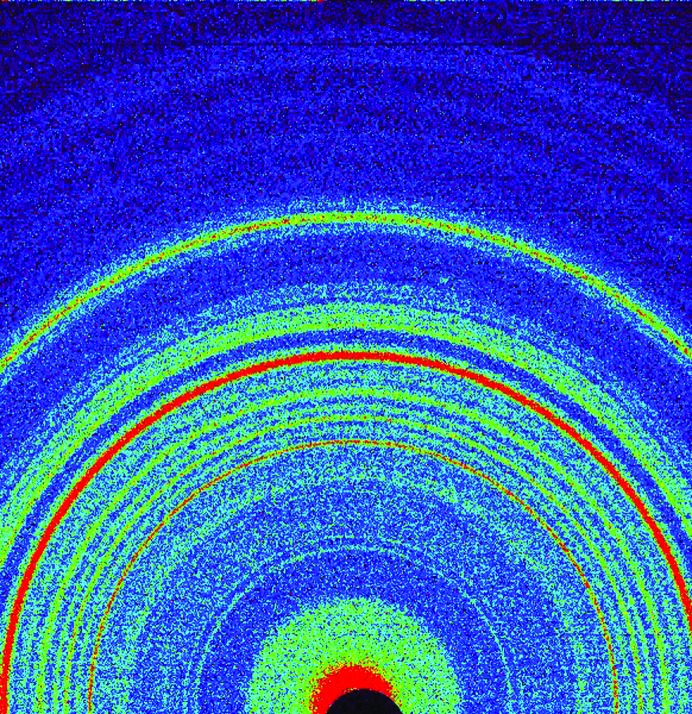
A colorized two-dimensional XRD pattern for the John Klein mudstone drill sample.

**Figure 9:**
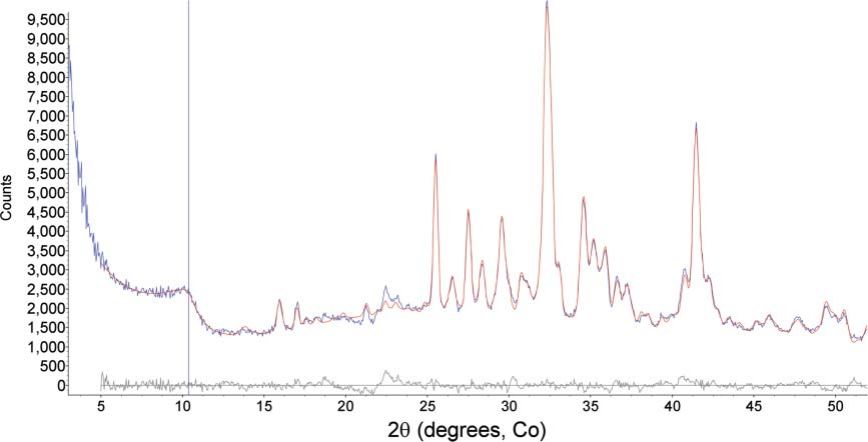
Rietveld refinement results for the John Klein mudstone (∼56.5 h integration). The vertical line near 10° 2θ represents the position of a Pearson VII profile used to model the ∼10 Å diffraction peak from a phyllosilicate. The differences near 22° 2θ are due to the 02*l* diffraction band from the phyllosilicate.

Two drill samples were subsequently obtained from adjacent locations called John Klein and Cumberland, from the Sheepbed mudstone member of the Yellowknife Bay formation (drilled on sols 182 and 279, respectively). This formation has been interpreted as a shallow lacustrine deposit by Grotzinger *et al.* (2014 ▶). Images of the drill holes for both samples and the Cumberland powder in the scoop are shown in Vaniman *et al.* (2014 ▶). Fig. 8 ▶ shows a colorized two-dimensional diffraction pattern for the John Klein sample; the most significant difference from the Rocknest data in Fig. 6 ▶ is the intensity just outside the beam stop, which is a reflection of low-angle diffraction intensity due to the presence of phyllosilicate(s) in the John Klein sample. Two-dimensional diffraction data for both samples were converted to one-dimensional patterns, and the data were analyzed using the Rietveld method (*TOPAS*) and *FULLPAT* (Figs. 9 ▶ and 10 ▶). Quantitative mineralogical results from Rietveld refinement and *FULLPAT* fitting for both Cumberland and John Klein are shown in Table 2 ▶. These data are similar in many respects to those obtained for Rocknest, revealing the presence of Fe-forsterite, plagioclase, pigeonite and augite. John Klein and Cumberland also contain orthopyroxene that was not found in Rocknest. The most important difference from the Rocknest mineralogy is the presence of phyllosilicate(s) in both mudstone samples, whereas no phyllo­silicate was detected in the Rocknest sample. In addition, John Klein and Cumberland contained much less ferromagnesian minerals such as Fe-forsterite, augite and pigeonite, and they both contained bassanite (CaSO_4_·0.5H_2_O) and considerably more magnetite.

The most striking differences in mineralogy between John Klein and Cumberland are the lack of Fe-forsterite in Cumberland and the presence in Cumberland of an expanded phyllosilicate, with *d*(001) near 14 Å, in addition to a broad poorly defined shoulder near 10 Å. Typical laboratory examinations of phyllosilicates such as vermiculite or smectite rely on a variety of treatments designed to facilitate discrimination between different minerals. These treatments include solvation with ethylene glycol, heat treatments, cation exchange and hydration treatments. With the possible exception of the last, none of these is available on Mars, making accurate discrimination between different phyllosilicates difficult. The relative humidity (RH) on Mars outside Curiosity varies from <1% at the warmest time of day to close to 100% at night (as shown by the appearance of frost on the surface). Maximum RH values in the vicinity of Curiosity ranged from ∼40–60% at ∼−65 to −75°C from late winter through to late spring, 2012–2013 (MSL Remote Environmental Monitoring Station data; http://photojournal.jpl.nasa.gov/catalog/PIA16915). Temperatures inside the body of the rover are consistently above 0°C, yielding much lower RH values, generally <1%. Given these conditions, the data in Figs. 9 ▶ and 10 ▶ are consistent with a collapsed clay mineral having a broad *d*(001) peak near 10 Å, and an expanded clay mineral with a broad *d*(001) peak near 14 Å. The breadth of these peaks eliminates any mica or well ordered chlorite from consideration. Furthermore, the breadth of the 001 reflections, coupled with evidence of an 02*l* diffraction band (from ∼22.5 to 23.1° 2θ), suggests that both the 10 and 14 Å phases are poorly ordered minerals similar to smectites. The position of the 02*l* diffraction band is consistent with a trioctahedral 2:1 phyllosilicate, such as saponite or Fe-saponite. No change in the position of the ∼14 Å peak was observed over at least 150 sols while the sample was held in the very low RH atmosphere inside CheMin (<1%), suggesting that the 14 Å phase is not a smectite with a hydrated (H_2_O-bearing) interlayer. Rather, the breadth of the 001 reflection, its position and the lack of change with time make it likely that this mineral represents a trioctahedral smectite containing chloritic interlayers that effectively ‘prop’ open the 2:1 layers (essentially a hydroxy pillared smectite). The pattern is not consistent with typical terrestrial vermiculites or chlorites. Although the presence of more highly charged interlayer cations such as Mg^+2^ or Ca^+2^ has been invoked as a possible explanation for the persistence of an expanded smectite interlayer (Vaniman *et al.*, 2014 ▶), the lack of change in *d*(001) with time at very low RH and the spacing of >12.5 Å (one H_2_O layer in the interlayer region) argue against this explanation (*e.g.* Bish *et al.*, 2003 ▶). The presence of poorly ordered phyllosilicates in both John Klein and Cumberland supports alteration involving liquid water, although the persistence of a significant amorphous component (∼30%) in both of these samples suggests either that interactions with liquid water were not long lived or that the hydrous minerals in these samples formed elsewhere and were later transported.

The presence of phyllosilicate(s) in Cumberland and John Klein, coupled with the increase in magnetite and decrease in Fe-forsterite relative to Rocknest, suggest that these trends may be related. Indeed, Vaniman *et al.* (2014 ▶) suggested that Fe-forsterite may have been consumed in a reaction that formed the phyllosilicate and magnetite, and similar reactions have been postulated for Martian meteorites. Bristow *et al.* (2014 ▶) presented a detailed discussion of the clay mineralogy in Cumberland and John Klein, potential alteration reactions and mass-balance calculations. They concluded that clay mineral formation occurred under surficial temperatures at circum-neutral pH, over a period of thousands to hundreds of thousands of years (based on Fe-forsterite alteration kinetics). These conclusions suggest the persistence of a potentially habitable environment, with oxidation of Fe^2+^ to Fe^3+^ available as a potential energy source. More detailed comparisons of the magnetite and Fe-forsterite in Rocknest with that in John Klein and Cumberland (*e.g.* unit-cell parameters and compositions) may shed light on these potential reactions, as will further mass-balance calculations using bulk chemistry and individual phase compositions derived from unit-cell parameters. These results illustrate the power of quantitative mineralogical information in understanding the past history of Mars and in inferring past formation environments. The detailed information obtained for these samples, including quantitative mineralogy, individual phase compositions from unit-cell parameters, clay mineralogy, and amorphous phase abundance and composition, could not have been obtained without diffraction data.

## 4. Conclusions and further work   

The miniaturized XRD/XRF instrument CheMin has excelled on Mars, returning diffraction data comparable in many respects with data available on Earth. This has been done remotely, using an instrument package approximately the size of a shoe box, with minimal sample preparation. The data obtained to date have already provided new insights into processes on Mars, and the instrument promises to return data that will answer numerous questions and clarify the past history of Gale crater. As mentioned above, several aspects of the instrument limit the quality of the XRD data, notably the comparatively large FWHM and the restricted angular range. Both of these could be improved through judicious modification of the instrument geometry, but they would likely necessitate an increase in size. Clay mineral identification will continue to be difficult due to the lack of appropriate treatments, although the CheMin team has already made use of analysis as a function of time, while a sample is held in the very low RH atmosphere of the CheMin instrument. Traditionally, one of the most significant issues limiting remote operation is the requirement for powder XRD of a finely powdered sample. CheMin largely surmounted this difficulty through the use of its unique sample vibration device. Ultimately, a contact XRD instrument would prove most useful for rapid analysis requiring no sample preparation; such an instrument has already been designed for the non-destructive analysis of works of art (Sarrazin, Chiari & Gailhanou, 2009 ▶). Although such an instrument using reflection geometry has significant drawbacks when examining macrocrystalline and polycrystalline materials, when perfected it would prove to be a powerful tool for the remote study of planetary bodies. A modification of this instrument potentially solves some of the difficulties by using a reflection geometry that combines Laue diffraction with energy-dispersive CCD detectors (Sarrazin, Dera *et al.*, 2009 ▶). This approach, coupled with data-processing software interfaced to the American Mineralogist Crystal Structure Database, may be the model for future remote contact diffraction analyses of the surfaces of planetary bodies.

## Figures and Tables

**Figure 10:**
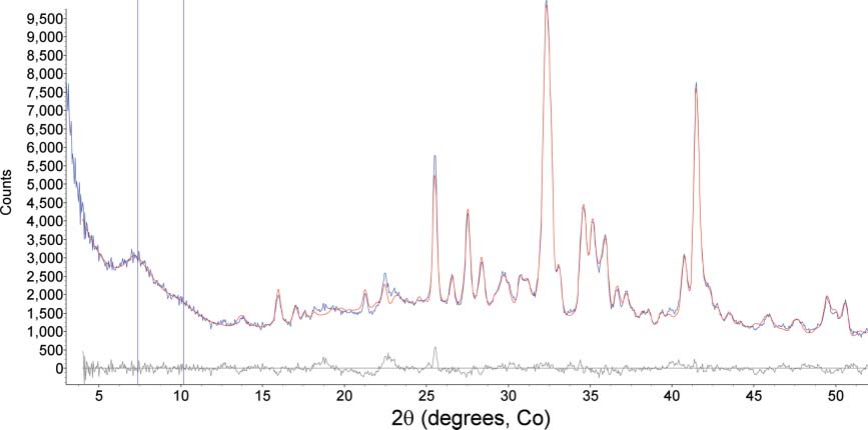
Rietveld refinement results for the Cumberland mudstone (∼41.1 h integration). The vertical lines near 7.4 and 10.1° 2θ represent the positions of split Pearson VII profiles used to model the ∼10 Å and ∼14 Å diffraction peaks from phyllosilicates. The differences near 22° 2θ are due to the 02*l* diffraction band from the phyllosilicates.

**Table 1:** Mineralogy of the Rocknest scoop 5 soil

Mineral	Weight%
Plagioclase	30
Forsterite-Fe	16
Augite	11
Pigeonite	10
Magnetite	1.5
Anhydrite	1.1
Quartz	1.0
Sanidine†	1.0
Hematite†	0.8
Ilmenite†	0.9
Amorphous	27

† At or near the detection limit.

**Table 2:** Mineralogy of the John Klein and Cumberland mudstones (wt%) With the exception of values 1%, all values have been rounded to the nearest unit.

Mineral	John Klein	Cumberland
Plagioclase	22	22
Fe-forsterite	3	1
Augite	4	4
Pigeonite	6	8
Orthopyroxene	3	4
Magnetite	4	4
Anhydrite	3	1
Bassanite	1	1
Sanidine	1	2
Quartz	0.4†	0.1†
Hematite	0.6†	1
Ilmenite		0.5†
Akaganeite	1	2
Pyrite	0.3†	
Pyrrhotite	1	1
Phyllosilicate	22	18
Amorphous	28	31

† At or near the detection limit.
